# Autoantibodies neutralizing type I interferons in older twin pairs: Low concordance

**DOI:** 10.70962/jhi.20260142

**Published:** 2026-10-09

**Authors:** Signe Bedsted Clemmensen, Nanna Mørk, Julie A. Jensen, Lisbeth Aagaard Larsen, Bettina Bundgaard, Marianne Nygaard, Michelle Møhlenberg, Morten K. Skouboe, Adrian Gervais, Paul Bastard, Anne Puel, Aurelie Cobat, Jean-Laurent Casanova, Kaare Christensen, Trine H. Mogensen

**Affiliations:** 1 Epidemiology, Biostatistics and Biodemography, Department of Public Health, University of Southern Denmark, Odense, Denmark; 2 The Danish Twin Registry, Department of Public Health, University of Southern Denmark, Odense, Denmark; 3 Department of Biomedicine, Aarhus University, Aarhus, Denmark; 4 Department of Infectious Diseases, Aarhus University Hospital, Aarhus, Denmark; 5 Laboratory of Human Genetics of Infectious Diseases, Necker Branch, Institut National de la Santé et de la Recherche Médicale U1163, Necker Hospital for Sick Children, Paris, France; 6 Imagine Institute, Paris Cité University, Paris, France; 7 Laboratory of Human Genetics of Infectious Diseases, Center for the Genetics of Host Defense, University of Texas Southwestern, Dallas, TX, USA; 8 Departments of Pediatrics and Immunology, and Children Research Institute, University of Texas Southwestern, Dallas, TX, USA; 9 Howard Hughes Medical Institute, New York, NY, USA; 10 Pediatric Hematology-Immunology and Rheumatology Unit, Necker Hospital for Sick Children, Assistance Publique-Hôpitaux de Paris, Paris, France

## Abstract

Autoantibodies (auto-Abs) neutralizing type I interferons (IFN-I) underlie various life-threatening viral infections, and the prevalence of these auto-Abs rises sharply between 65 and 70 years of age. We investigated the familial aggregation of neutralizing IFN-I auto-Abs by assessing concordance rates in Danish older twins, including 3,270 twins aged 65–96 years in a sample comprising 1,318 intact twin pairs (2,636 individuals) and 634 twins from broken twin pairs (634 individuals). Neutralizing IFN-I auto-Abs were detected in 1.5% (50/3,270) of all twins tested, including 1.4% (37/2,636) in twins from intact pairs and 2.1% (13/634) in twins from broken pairs. A total of 50 twins had neutralizing IFN-I auto-Abs, including one concordant monozygotic pair and 35 discordant pairs, yielding a low overall concordance rate. A slight, but nonsignificant, increase in mortality was associated with harboring neutralizing IFN-I auto-Abs. Collectively, these findings do not reveal any familial aggregation of IFN-I auto-Abs, arguing against a strong germline genetic etiology of IFN-I auto-Abs in older adults.

## Teaser

Here, Clemmensen et al. investigated the heredity of IFN-I auto-Abs in adults in a large Danish twin cohort of 1,318 twin pairs. Overall, the data do not support a strong familial aggregation, as reflected by a low concordance rate for IFN-I auto-Abs, suggesting that development of auto-Abs in older individuals is predominantly mediated by age-related immunological mechanisms.

## Introduction

Type I interferons (IFN-I) constitute a first line of immunity against viral infections (1). Autoantibodies (auto-Abs) neutralizing IFN-I have emerged as global, common, and strong determinants of a growing number and widening range of severe viral diseases. Neutralizing anti-IFN-I auto-Abs (hereafter referred to as IFN-I auto-Abs) were initially described in the 1980s in a patient with disseminated varicella (2, 3), but they were long thought to be clinically silent. Their status changed in 2020, with the discovery that pre-existing auto-Abs neutralizing IFN-I account for about 10% of critical COVID-19 cases in unvaccinated adults and are present in an even higher fraction of cases of fatal COVID-19 (4, 5, 6). Subsequently, IFN-I auto-Abs were found to also underlie other severe respiratory infections, including 5% of cases of critical influenza pneumonia (7), a fatal case of H5N1 avian influenza (8), and 20% of cases of critical Middle Easter respiratory syndrome (MERS) pneumonia (9). These IFN-I auto-Abs have also been found to underlie viral infections in the central nervous system, including about 40% of cases of West Nile virus (WNV) encephalitis (10, 11), 10% of cases of tick-borne virus encephalitis (TBE) (12), and severe cases of other types of arbovirus encephalitis, such as Powassan encephalitis, Usutu encephalitis, and Ross River disease (13). Another study showed that auto-Abs against IFN-I underlie more than a third of HSV-1–triggered fulminant viral hepatitis (14). Finally, IFN-I auto-Abs have been associated with adverse reactions to the yellow fever live-attenuated vaccine (15) and chikungunya virus live-attenuated vaccine (VLA1553) (16). Overall, IFN-I auto-Abs can underlie a significant number of cases of life-threatening viral diseases in adults. More recent studies have linked the presence of IFN-I auto-Abs to the severity of rheumatological conditions (17, 18) and found them to be a potential modifying risk factor for cancer development, whereas exerting a beneficial effect on the response to immunotherapy (19, 20), sparking an interest in investigating a broader role of IFN-I auto-Abs in the pathogenesis of various autoimmune, inflammatory, and malignant conditions (21).

As to the origin of IFN-I auto-Abs, these have been reported in rare inborn errors of immunity (IEI) affecting thymic tolerance. These include patients with autocrine autoimmune polyglandular polyendocrine syndrome type I due to deleterious variants of AIRE (22, 23), immune-dysregulation poly-endocrinopathy enteropathy X-linked caused by deleterious variants of FOXP3 (24), and combined immunodeficiency with biallelic hypomorphic RAG1 or RAG2 variants (25). More recently, IFN-I auto-Abs were associated with defects in alternative NF-κB signaling, including IEI in autosomal recessive NIK or RelB deficiency (26), as well as in women with X-linked incontinentia pigmenti (27). These findings collectively suggest a germline inheritance in these rare, mostly childhood diseases.

A seminal study on a large French general population–based cohort of ∼34,000 individuals found that the prevalence of circulating auto-Abs neutralizing IFN-α2 and/or IFN-ω is low (about 0.3% for auto-Abs neutralizing high concentrations of IFN-I and 1% for auto-Abs neutralizing low concentrations of IFN-I) and remains largely stable until the age of 65 years (5, 6). Remarkably, after the age of 65, the prevalence of neutralizing IFN-I auto-Abs increases sharply, reaching up to 4% for low concentrations of IFN-I and 7% for high concentrations of IFN-I in individuals aged 80–85 years (6). A less pronounced age-related increase was observed in 1,876 antiretroviral therapy-treated individuals aged 65–94 years from the Swiss HIV Cohort Study, where prevalence of IFN-I auto-Abs reached 1.7% in the 70–79-year-olds (28). However, whether the high frequency in individuals above 65 years of age has a genetic component and whether this relates to germline or somatic genetic variants remains unresolved. Here, we used a Danish population-based study of 3,270 twins aged 65–96 years with more than a decade of mortality follow-up to investigate whether IFN-I auto-Abs show familial aggregation in a population-based sample.

## Results

### Neutralizing IFN-I auto-Abs in intact and broken twin pairs

**Figure 1.**
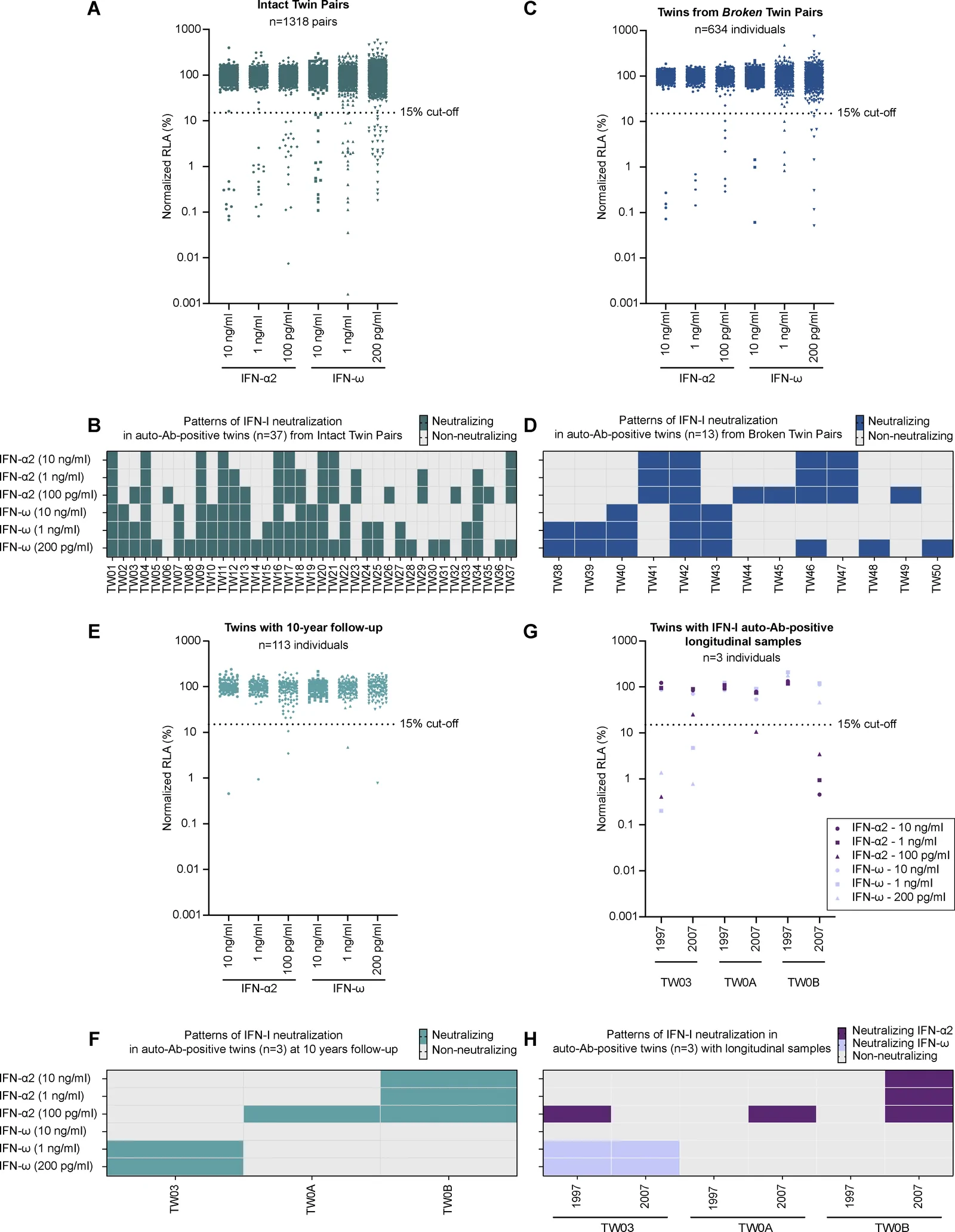
**Capacity of plasma samples to neutralize IFN-I. (A, B, and E)** Plasma samples from 1,318 intact twin pairs (*n* = 2,636 individuals) and 634 twins from broken twin pairs were tested for their ability to neutralize at different IFN-I stimuli applied to HEK cells for 16 h. Neutralization was defined as normalized RLA (%) below 15% of the median induction ratio, based on previous data from the general population showing this cutoff value as the best for separating the population into two groups (6), and represented as a dotted line in each panel. All samples were tested once per IFN-I condition due to the limited amount of material. **(C, D, and G)** Heatmaps showing patterns of IFN-I neutralization among IFN-I auto-Ab–positive twins. Each column represents samples from one individual, and each row represents a specific IFN-I condition. Dark/colored indicates neutralizing activity, and light grey indicates non-neutralizing activity. **(A and C)** Neutralizing activity against IFN-α2 and IFN-ω at high (10 ng/ml), intermediate (1 ng/ml), and low (100 pg/ml and 200 pg/ml, respectively) IFN-I concentrations in plasma samples from 1,318 intact twin pairs (A) and patterns of IFN-I neutralization in IFN-I auto-Ab–positive twins (*n* = 37) from intact twin pairs (TW01–TW37) (C). **(B and D)** Neutralizing activity against the same IFN conditions as mentioned in A in plasma samples from 634 twins from broken twin pairs (B) and patterns of IFN-I neutralization in IFN-I auto-Ab–positive twins (*n* = 13) from broken twin pairs (TW38–TW50) (D). **(E)** Neutralizing activity against the same IFN conditions as mentioned in A in plasma samples from 113 of the twins from the intact twin pairs who had a plasma sample drawn 10 years later. **(F)** Neutralizing activity against the same IFN conditions as mentioned in A in the three twins from E and G who were IFN-I auto-Abs positive in their 10-year follow-up sample, plotted with the neutralization results in the original sample 10 years prior. Dark purple represents stimulation with IFN-α2 and light purple IFN-ω. **(G)** Patterns of IFN-I neutralization in IFN-I auto-Ab–positive twins (*n* = 3) from intact twin pairs with a 10-year follow-up sample (TW03, TWA0, and TWB0). **(H)** Patterns of IFN-I neutralization in the twins from F showing persistence of neutralizing IFN-I auto-Abs in one twin (TW03) and development of neutralizing IFN-I auto-Abs in the two other twins (TW0A and TW0B). Dark purple indicates neutralization activity against IFN-α2, light purple indicates neutralization activity against IFN-ω, and light grey color indicates no neutralizing activity.

A total of 1,318 intact twin pairs (1,013 twin pairs aged 65–80 from the Middle Age Danish Twins [MADT]/Middle age Danish Twins study [MIDT] cohort and 305 twin pairs aged 73–94 from the Longitudinal Study of Aging Danish Twins [LSADT] cohort) were available for measurement of IFN-I auto-Abs in a HEK-based luciferase assay as previously done (4, 6). Measurements were prioritized for IFN-α2 and IFN-ω, the main targets of neutralizing auto-Abs (6). Neutralization was defined as below 15% of the median of the normalized relative luciferase activity (RLA), based on a previous study of 3,219 samples from the general population, which demonstrated 15% as the best cutoff value, separating the population into two groups (6). An additional 634 twins from broken twin pairs (34 twins aged 76–96 from the LSADT cohort and 600 twins aged 65–80 from the MADT/MIDT cohort) were also available for measurement of IFN-I auto-Abs. Among the 1,318 intact twin pairs, the median age at blood sampling was 71.4 years, and 29% of pairs were monozygotic (Table 1). Blood samples within twin pairs were collected with a median difference of 21 days. Among these individuals, 37/2636 were IFN-I auto-Abs positive, corresponding to a prevalence of 1.4% (Fig. 1, A and C, and Table S1). Further descriptive characteristics, including cohort-specific descriptives for LSADT and MADT/MIDT, are shown in Table 1. Among the 634 twins from broken twin pairs, the median age at blood sampling was 72.0 years, and 15% of the pairs were monozygotic. Among these individuals, 13/634 were IFN-I auto-Abs positive, corresponding to a prevalence of 2.1% (Fig. 1, B and D, and Table S1). The prevalence of 2.1% among twins from broken twin pairs was slightly higher (P = 0.23) than the prevalence of 1.4% among twins from intact pairs. In a small subset of twins from intact pairs (*n* = 113), all from the LSADT cohort from 1997, we had access to plasma samples taken 10 years later (2007). Three out of these 113 twins (2.7%) were IFN-I auto-Abs positive (Fig. 1, E and G, and Table S1). Only one of these three was IFN-I auto-Abs positive in the first sample taken 10 years earlier, whereas the other two twins did not have detectable neutralizing IFN-I auto-Abs 10 years earlier, but instead had developed neutralization over these 10 years (Fig. 1, F and H).

**Table 1.** Characteristics of the sample of intact twin pairs

​	LSADT	MADT/MIDT	Total
Birth cohorts	1902–1923	1931–1946	1902–1946
Total number of individuals	610	2,026	2,636
Females, *n* (%)	409 (67%)	1,006 (50%)	1,415 (54%)
Monozygotic, *n* (%)	252 (41%)	514 (25%)	766 (29%)
Same-sex dizygotic, *n* (%)	352 (58%)	744 (37%)	1,096 (42%)
Opposite-sex dizygotic, *n* (%)	6 (1%)	768 (38%)	774 (29%)
Age at blood sampling in years, median (range)	77.5 (73.2–94.3)	69.3 (65.0–79.9)	71.4 (65.0–94.3)
IFN-I auto-Abs positive, *n* (%)	9 (1.5%)	28 (1.4%)	37 (1.4%)

LSADT, Longitudinal Study of Aging Danish Twins; MADT/MIDT, Middle-aged Danish Twin study.

### Comparison of the prevalence of neutralizing IFN-I auto-Abs in a cohort of Danish twins and a French general population–based cohort

**Figure 2.**
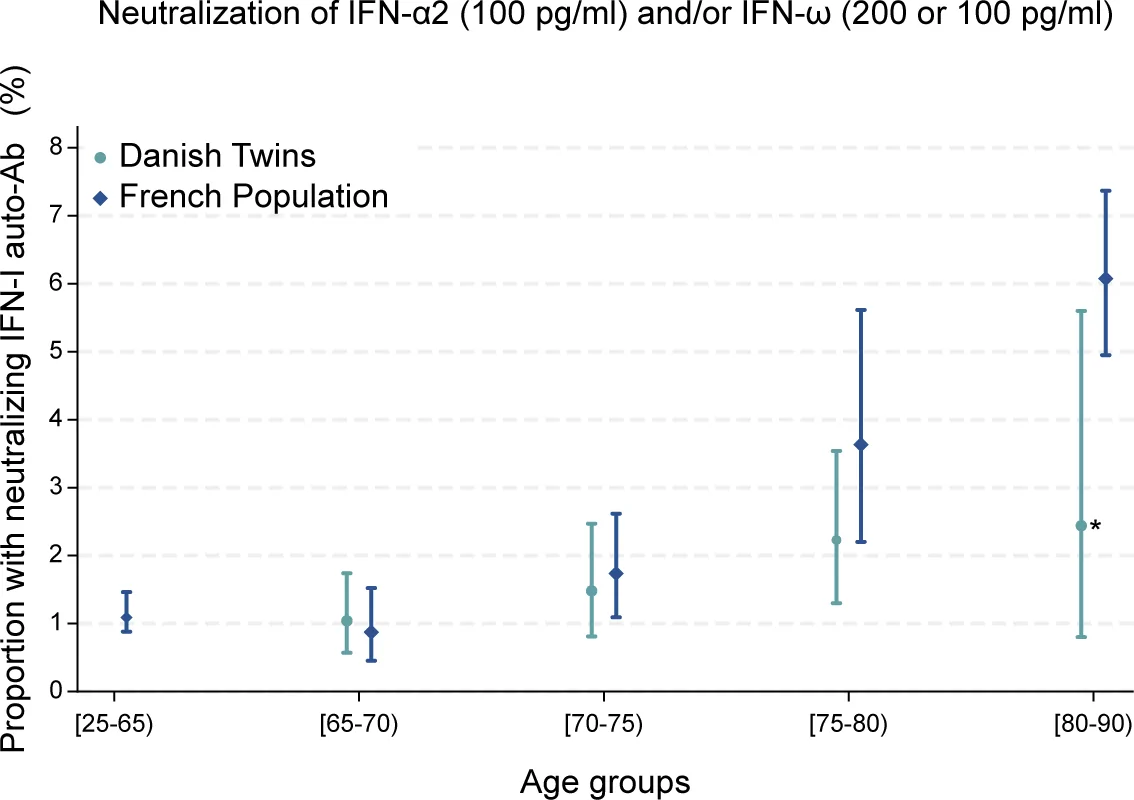
**Comparison of the prevalence of neutralizing IFN-I auto-Abs in Danish twins compared with the French general population.** Comparison of the prevalence of neutralizing IFN-I auto-Abs in the Danish twins (both twins from intact twin pairs and twins from broken twin pairs) and a subgroup of 10,778 French individuals from a previously published French general population–based cohort (6). Green circular markers represent Danish twins, and blue diamond markers represent the French general population–based cohort; vertical lines represent 95% CI. Y-axis is the proportion of individuals with neutralizing IFN-I auto-Abs (%), and the x-axis is age groups (years). Represents age intervals; Square brackets indicate that the age is included, whereas parentheses indicate that the age is excluded. We pooled and plotted data from the [25–65)-year-olds from the French general population–based cohort since we do not have measurements from Danish twins <65 years old. We also pooled data from the [80–90)-year-olds from the French general population-based cohort to compare with the oldest Danish twins from the same age group. Twins >90 years old are not included in this comparison due to the few individuals in total and no IFN-I auto-Ab–positive individuals. There is no significant difference in the prevalence of IFN-I auto-Abs in Danish Twins and the French Population in the age groups [65–70), [70–75), and [75–80), but in the age group [80–90) there is a significantly lower prevalence of IFN-I auto-Abs in the Danish twins (marked with the *) compared with the French general population–based cohort.

We next compared the prevalence of neutralizing IFN-I auto-Abs in the Danish twins with a previously published French general population–based cohort (6). We compared the Danish twins to a subgroup of 10,778 French individuals in this cohort who had samples measured for neutralization against both IFN-α2 and IFN-ω at 100 pg/ml in a similar HEK-based luciferase assay. All the IFN-I auto-Ab–positive twins in our study were able to neutralize at least IFN-α2 and/or IFN-ω at 100 pg/ml or 200 pg/ml, respectively (Fig. 1, A–D), and we therefore chose to compare the prevalence of neutralizing IFN-I auto-Abs in the Danish twins with the prevalence of neutralizing auto-Abs against IFN-α2 and/or IFN-ω at 100 pg/ml in the French general population. Both twins from the intact twin pairs and the broken twin pairs are included in this analysis. In our comparison, the prevalence of IFN-I auto-Abs did not significantly differ between the Danish twins and the French general population–based cohort, except for the oldest twins aged [80–90) years, where we found a significantly lower prevalence of IFN-I auto-Abs in the Danish twins (P = 0.04) (Fig. 2). Of note, this difference in prevalence among the oldest age groups between the two cohorts may partly reflect the difference in concentrations used for measuring neutralizing activity against IFN-ω (200 pg/ml in the present study of Danish twins vs. 100 pg/ml in in the French general population–based cohort), potentially resulting in a slight underestimation of the IFN-I auto-Abs prevalence in the Danish twin cohort.

### Low concordance for neutralizing IFN-I auto-Abs in intact Danish twin pairs

Among the 1,318 intact twin pairs, one concordant monozygotic twin pair was found, yielding an estimated monozygotic proband-wise concordance rate of 17% (Table 2). Prevalence was similar across zygosity groups. Given the observed individual prevalence of neutralizing IFN-I auto-Abs (1.4%), the expected number of concordant twin pairs under independence was ∼0.3 (i.e., less than one pair on average). The finding of one concordant pair among 1,318 twin pairs corresponds to an estimated 23% probability of observing at least one concordant pair assuming within-pair independence. Among the 383 monozygotic twin pairs, the observed prevalence was 1.6%, corresponding to ∼0.1 expected concordant monozygotic pairs and a 9% probability of observing at least one concordant monozygotic pair under independence (Table 2).

**Table 2.** Number of concordant and discordant pairs, prevalence, and proband-wise concordance rate for IFN-I auto-Abs by zygosity in the sample of intact twin pairs

IFN-I auto-Abs	Monozygotic	Same-sex dizygotic	Opposite-sex dizygotic	Total
Concordant pairs, *n*	1	0	0	1
Discordant pairs, *n*	10	13	12	35
IFN-I auto-Abs prevelence	1.6%	1.2%	1.6%	1.4%
Concordance rate (95% CI)	17% (0.5–58%)	-	-	5.4% (0.1–16%)
Expected concordant pairs^a^	0.1	0.1	0.1	0.3
Probability of observing at least one concordant pair^a^	9.3%	7.6%	9.4%	23%

Note: A concordant pair includes two affected individuals, whereas a discordant pair includes one affected individual.

a Calculated assuming within-pair independence.

### Mortality in twin pairs discordant for neutralizing IFN-I auto-Abs

The main mortality analyses were based on IFN-I auto-Ab–positive twins from discordant twin pairs, their IFN-I auto-Ab–negative co-twin, and their matched controls. Descriptive characteristics of these groups, including lifestyle factors, and follow-up information are shown in Table 3. The IFN-I auto-Ab–positive twins lived a median of 23 days shorter since blood sampling than their IFN-I auto-Ab–negative co-twin. In Cox models, no clear difference in mortality was observed for IFN-I auto-Ab–positive twins compared with their matched controls (adjusted hazard ratio [HR]: 1.08, 95% confidence interval [CI]: 0.61–1.92) nor for IFN-I auto-Ab–negative co-twins compared with their matched controls (adjusted HR: 1.24, 95% CI: 0.71–2.17), with similar estimates in unadjusted models (Table 4). Mortality showed a tendency to be higher among IFN-I auto-Ab–positive twins than among their negative co-twins, although there was no statistical evidence of a difference (adjusted HR: 1.28, 95% CI: 0.57–2.87). For context, these HRs were smaller than the association between male and female mortality, with higher mortality among males, both in the sample of matched controls (*n* = 280) (adjusted HR: 1.53, 95% CI: 1.06–2.20) and in the full sample of intact twin pairs (*n* = 2,636) (adjusted HR: 1.44, 95% CI: 1.26–1.64). The proportional hazards assumption held for the main exposure across all models. Some secondary covariates showed evidence of non-proportionality, and the global test was statistically significant in the sex mortality models (P = 0.04 for the model in matched controls and P < 0.001 in the full sample).

**Table 3.** Characteristics of IFN-I auto-Ab–positive twins and their negative co-twins from intact twin pairs and matched controls

​	IFN-I auto-Ab–positive twins	IFN-I auto-Ab–negative co-twins^a^	Matched controls for IFN-I auto-Ab–positive twins	Matched controls for IFN-I auto-Ab–negative twins
*N*	35	35	140	140
Females, *n* (%)	12 (34%)	16 (46%)	48 (34%)	64 (46%)
**Zygosity**	​	​	​	​
Monozygotic, *n* (%)	10 (29%)	10 (29%)	44 (31%)	48 (34%)
Same-sex dizygotic, *n* (%)	13 (37%)	13 (37%)	58 (41%)	58 (41%)
Opposite-sex dizygotic, *n* (%)	12 (34%)	12 (34%)	38 (27%)	34 (24%)
Age in years at time of blood sampling, median (range)	73.6 (66.7–86.5)	73.6 (66.8–86.3)	73.6 (66.7–86.5)	73.6 (66.8–86.3)
Total observed time, years	373	406	1,559	1,606
Median observed time year (range)	12.3 (1.2–15.5)	13.8 (0.1–20.8)	13.3 (0.7–21.2)	13.3 (0.0–23.3)
Deaths, *n* (%)	23 (66%)	21 (60%)	81 (58%)	75 (54%)
BMI, mean (SD)	26.9 (4.8)	26.3 (3.7)	26.7 (4.2) (3 missing)	26.4 (3.8) (1 missing)
Education (higher education), *n* (%)	8 (23%) (3 missing)	11 (31%) (1 missing)	38 (27%) (8 missing)	29 (21%) (7 missing)
Alcohol consumption (drinker), *n* (%)	32 (91%)	32 (91%)	125 (89%) (1 missing)	132 (94%) (1 missing)
Smoking (smoker), *n* (%)	24 (69%)	19 (54%)	89 (64%) (2 missing)	87 (62%) (1 missing)

a Co-twin refers to the other twin from the same intact twin pair as the IFN-I auto-Ab–positive twin.

**Table 4.** HRs and 95 % confidence intervals for IFN-I auto-Abs status

​	Basic model HR (95 % CI)	Adjusted model HR (95% CI)
IFN-I auto-Ab–positive twins vs. matched controls	1.16 (0.69–1.98)	1.08 (0.61–1.92)^a^
IFN-I auto-Ab–negative co-twins^c^ vs. matched controls	1.17 (0.68–1.99)	1.24 (0.71–2.17)^a^
IFN-I auto-Ab–positive twins vs. negative co-twins^c^	1.36 (0.63–2.97)	1.28 (0.57–2.87)^b^

a Adjusted for BMI, education, alcohol consumption, and smoking.

b Adjusted for BMI and smoking.

c Co-twin refers to the other twin from the same intact twin pair as the IFN-I auto-Ab–positive twin.

### Mortality in intact vs. broken twin pairs

Finally, mortality was higher among the twins from broken pairs compared with twins from intact pairs, irrespective of IFN-I auto-Abs status (unadjusted model: HR = 1.17, 95% CI 1.03–1.33, adjusted model: HR = 1.17, 95% CI: 1.02–1.34), as expected based on a series of Danish twin studies demonstrating a substantial genetic component to lifespan and hence an increased mortality risk among twins with a deceased co-twin (29, 30, 31). The proportional hazards assumption was met for pair type (intact vs. broken), but some covariates showed non-proportionality, and the global test was statistically significant (P < 0.001), likely reflecting age-related changes in mortality over time. Descriptive characteristics, including lifestyle factors, and follow-up information of twins from intact (*n* = 2,636) and broken (*n* = 634) twin pairs are presented in Table S2.

### Mortality in twins during the COVID-19 pandemic

At the beginning of the COVID-19 pandemic period in Denmark (February 1, 2020 to November 1, 2022), 29 IFN-I auto-Ab–positive twins from intact and broken pairs in MADT/MIDT were alive, while all from the older LSADT cohort were dead. In Cox models, IFN-I auto-Ab status was not significantly associated with mortality during COVID-19. Among all twins alive at the start of the COVID-19 period (from intact and broken pairs), the unadjusted HR was 1.36 (95% CI: 0.61–3.05) and the adjusted HR was 1.18 (95% CI: 0.54–2.61), which is very similar to the estimate for the whole study period. There were no violations of the proportional hazards assumption in these models.

## Discussion

In this study, we investigated whether the presence of neutralizing IFN-I auto-Abs in older individuals is genetically driven by determining the prevalence of these auto-Abs in 1,318 intact twin pairs and 634 twins from broken twin pairs. Overall, we found a prevalence of 1.4% (37/2636) IFN-I auto-Ab–positive twins within the group of intact twin pairs and a non-statistically significant higher prevalence of 2.1% (13/634) in twins from broken twin pairs. Among the 1,318 intact twin pairs, there was one concordant monozygotic twin pair, yielding an estimated monozygotic proband-wise concordance rate of 17% compared with the chance of finding at least one concordant pair by random of 23% with the number of twin pairs and IFN-I auto-Abs prevalence of the present study. Among the 383 monozygotic twin pairs, ∼0.1 concordant pairs would be expected under within-pair independence, corresponding to a 9% chance of observing at least one concordant monozygotic pair. Although this observation may suggest a modest degree of familial aggregation, we were not formally able to detect a genetic predisposition to neutralizing IFN-I auto-Abs in this large cohort of twins older than 65 years.

We also investigated the association between IFN-I auto-Abs and mortality in the discordant twins from the intact twin pairs and found a modest, but statistically nonsignificant, increase in mortality risk among IFN-I auto-Ab–positive twins.

The absence of a highly significant concordance or familial aggregation for germline genetics as determining the presence of IFN-I auto-Abs may not be surprising, given the current evidence from familial studies and IEI. Such studies have led to the identification of only a few selected monogenic defects in AIRE, FOXP3, RAG1/2, and alternative NF-κB signaling, underlying IFN-I auto-Abs in infants and children (22, 23, 24, 25, 26, 27), and none for IFN-I auto-Abs emerging in older individuals. Although some monogenic diseases, such as Huntington’s chorea (32) and autosomal dominant Alzheimer’s disease (33), may not present until middle or late life, the relatively high frequency of IFN-I auto-Abs in older individuals, rising to 4–7% in people aged over 65 years (5, 6), does not lend strong support to monogenic defects or genetic homogeneity underlying the development of IFN-I auto-Abs. Instead, the emergence of neutralizing IFN-I auto-Abs may be caused by the combined effect of several age-related immunological and biological processes, similar to clonal hematopoiesis (34) emerging in a considerable proportion of individuals older than 65 without any clear familial aggregation. Despite the absence of a strong germline genetic link, at least not when assessed through a familial aggregation study, there is likely pathophysiological homogeneity, i.e., unifying biological mechanisms underlying the emergence of IFN-I auto-Abs in infants and children as well as in older individuals. Thymic involution in older individuals appears as a biological and immunological factor, which has been previously proposed to contribute to the increased prevalence of IFN-I auto-Abs (35, 36). Indeed, several studies have suggested a considerable variability in age-related thymic involution (37, 38). Moreover, IFN-I–specific B cells were recently shown to be present before severe viral disease (COVID-19) in IFN-I auto-Ab–positive individuals, supporting the idea that the age-related increase in IFN-I auto-Abs prevalence is caused by thymic decline impairing central tolerance (39). Collectively, our data, together with other studies, are consistent with the idea that impaired thymic function contributes to the development of defective central tolerance and IFN-I auto-Abs production, which in infants and children may be caused by specific genetic defects and in older adults by various other biological and environmental factors (36).

The finding of lack of a clear genetic component to IFN-I auto-Abs development should be interpreted with caution, because, despite the large sample size, statistical power was limited due to the low prevalence of the trait in question, i.e., IFN-I auto-Abs. The observation of one concordant monozygotic pair despite only ∼0.1 expected concordant monozygotic pairs suggests that a modest degree of familial aggregation cannot be excluded. Moreover, the mortality analyses should be interpreted with caution given the limited number of IFN-I auto-Ab–positive individuals. It is possible that a larger sample size and/or a population with a higher frequency of IFN-I auto-Abs might have revealed a higher degree of concordance, reflecting more familial aggregation.

One factor that renders a study of IFN-I auto-Abs difficult in older twins is the dynamics of emergence and disappearance of IFN-I auto-Ab–positive individuals over time, i.e., as increasing fractions of individuals develop auto-Abs while others are dying with or from these IFN-I auto-Abs from severe infection or other IFN-I auto-Ab–associated causes. Our findings of a slight and statistically nonsignificant higher prevalence of IFN-I auto-Abs in twins from broken pairs compared with twins from intact twin pairs argue against a high IFN-I auto-Ab–associated mortality and render this issue unlikely to explain the lack of concordance in this study. These data, together with the relatively low prevalence of IFN-I auto-Abs in the general population, may argue for screening approaches targeting particular high-risk groups of susceptible individuals, rather than the entire population.

The presence of neutralizing IFN-I auto-Abs has been demonstrated to underlie a wide range of severe viral infections in lungs (including fatal COVID-19), brain, skin, and liver (5, 6); fatal H5N1 avian influenza (8); fulminant herpes simplex virus hepatitis (14); MERS pneumonia and influenza; WNV and TBE; and other rare arboviral types of encephalitis (9, 10, 11, 13); as well as severe reactions to yellow fever and chikungunya live-attenuated vaccines (15, 16). In the present study, we found that mortality was higher in twins with neutralizing IFN-I auto-Abs compared with their IFN-I auto-Ab–negative co-twin, although this difference was modest and statistically non-significant. We did not observe any association between IFN-I auto-Abs status and mortality during COVID-19 in our twin cohorts, possibly because the observation period was limited and the number of twins who died during COVID-19 was relatively low. These findings suggest that harboring IFN-I auto-Abs in isolation may not be significantly associated with markedly increased mortality from all causes of mortality in the general population of older individuals, whereas such auto-Abs confer a major risk of severe viral infections in the lung and brain. Moreover, recent evidence suggests that together with comorbidities, immune senescence, systemic inflammation, and metabolic disturbances, these neutralizing IFN-I auto-Abs have a significant negative impact on survival, not only from viral infection but also from immunological diseases and cancer (17, 18, 19, 20). Finally, several diverse, potentially opposing roles of IFN-I auto-Abs in health and disease may exist. For example, a detrimental role in predisposition to fatal viral infections versus a beneficial role against cancer development and response to immunotherapy may obscure the complete picture of IFN-I auto-Abs and associations with mortality (19, 40).

In conclusion, despite a large, population-based twin sample with many years of follow-up, we could not detect a genetic predisposition to the development of neutralizing IFN-I auto-Abs in twins older than 65 years. The presence of IFN-I auto-Abs was associated with a slightly increased mortality, although this difference did not reach significance. These findings, together with other studies, are consistent with the idea that impaired thymic function contributes to the development of defective central tolerance and IFN-I auto-Abs production—which in infants and children may be caused by specific germline genetic lesions—and in older individuals to a higher extent by somatic factors, whether genetic or epigenetic. Future studies on the genetics and immune cell subsets, as well as larger longitudinal population samples and disease cohorts, together with more detailed epidemiological data and repeated sampling over time, are needed to achieve a deeper insight regarding the origin, emergence, and persistence of IFN-I auto-Abs in older individuals. In this context, longitudinal studies in middle-aged and older individuals assessing the development and persistence of IFN-I auto-Abs, and linking this to infection history, autoimmunity, cancer, and mortality, are warranted.

## Materials and methods

### Cohorts

This study included 3,272 twins recruited by the Danish Twin Registry (DTR) as part of three twin studies: the LSADT, the study of MADT, and the MIDT (41, 42). Briefly, LSADT is a cohort-sequential study of primarily same-sex twin pairs aged 70 years and older. It includes a total of 4,731 twins and was initiated in 1995 with follow-up assessments every second year through 2007 (43). The individuals included in the present study from LSADT all participated in the 1997 assessment and a subset in the 2007 assessment. In both assessments, blood samples were collected. MADT was initiated in 1998 and includes 4,314 twins randomly chosen from the birth years 1931–1952. Surviving participants were revisited from 2008 to 2011, when the blood samples used in this study were collected. MIDT was performed together with the follow-up wave of MADT from 2008 to 2011 and includes 10,276 twins born between 1931 and 1969. For the present study, participants from MADT and MIDT were selected among twins who were 65 years and older at blood sampling. This study comprises “intact twin pairs,” where we have a blood sample from both twins, and “twins from broken twin pairs,” where we have a blood sample from only one of the two twins, from pairs where the co-twin was deceased when the twin had the blood sample drawn after the age of 65 years.

Information on body mass index (BMI), smoking, alcohol consumption, and education was obtained from questionnaire data collected in the same assessment as the blood sampling. Information about vital status was obtained through the Danish Civil Registration System (44), and twins were followed until emigration, death, or the end of follow-up, August 1, 2024, whichever came first.

This study is based on plasma samples from 3,272 twins recruited by the DTR from the LSADT and MADT/MIDT cohorts. A total of 1,318 intact twin pairs (1,013 twin pairs aged 65–80 from the MADT/MIDT cohort and 305 twin pairs aged 73–94 from the LSADT cohort) were available for measurement of IFN-I auto-Abs. An additional 636 twins from broken twin pairs (34 twins aged 76–96 from the LSADT cohort and 602 twins aged 65–80 from the MADT/MIDT cohort) were also available for measurement of IFN-I auto-Abs. Plasma samples from two twins from broken twin pairs were excluded due to poor sample quality resulting in invalid luciferase signals; see below for further technical explanation. This yielded 3,270 plasma samples for analysis, resulting in 1,318 intact twin pairs and 634 twins from broken twin pairs.

For comparison of the prevalence of neutralizing IFN-I auto-Abs in the Danish twins with a background population, we used previously published data from a French general population–based cohort (6), where we compared our results with a subgroup of 10,778 French individuals in this cohort who had samples measured for neutralization against both IFN-α2 and IFN-ω at 100 pg/ml in a similar HEK-based luciferase assay as described below.

### IFN-I neutralization assay

The ability of plasma samples to neutralize IFN-I was assessed as previously described (6, 10). Briefly, plasma samples were heat-inactivated in a PCR cycler at 56°C for 20 min prior to use. HEK-293T cells were seeded in 96-well plates with 15,000 cells per well in 100 μl DMEM (#D6429; Sigma-Aldrich) supplemented with 10% FCS (#F7524; Sigma-Aldrich) and 1% penicillin-streptomycin (pen-strep) (#10378-016; Thermo Fisher Scientific, 100 IU/ml). After overnight incubation, cells were transfected with 98 ng pGL4.45 (ISRE firefly luciferase) and 2 ng of a *Renilla* luciferase-encoding plasmid per well using X-tremeGENE 9 DNA Transfection Reagent (#06365787001; Sigma-Aldrich) in OptiMEM (#31-985-070; Thermo Fischer Scientific), at a transfectant:DNA ratio of 1:3. The following day, the supernatant was removed, and cells were incubated for 16 h in 50 μl DMEM containing the indicated IFN-I (see below), 2% FCS, 1% pen-strep, and 10% plasma. Firefly and *Renilla* luciferase activities were measured using the Dual-Luciferase Reporter Assay System (#E2980; Promega). Unglycosylated IFN-I was tested at 10 ng/ml, 1 ng/ml, and 100 pg/ml for IFN-α2 (130-093-874; Miltenyi Biotec) and at 10 ng/ml, 1 ng/ml, and 200 pg/ml for IFN-ω (300-02j-100UG; PeproTech). Luminescence was measured using a Synergy LX multi-mode reader (BioTek). Samples with *Renilla* luciferase activity <18% of the plate median were excluded due to low transfection efficiency or blood contamination. RLA was calculated as firefly luciferase activity divided by *Renilla* luciferase activity. To remove the baseline signal, RLA values were further normalized to the RLA value of a control sample from the same plate not stimulated with IFN-I. This baseline-corrected RLA value was then normalized to the median induction level of the plate of non-neutralizing samples. The results are expressed as a percentage of the normalized RLA after baseline correction, hereafter referred to as normalized RLA (%). Due to the limited amount of material available for analysis, each plasma sample from a twin was analyzed one time per IFN-I condition. Samples were considered neutralizing if the normalized RLA (%) was below 15% of the median control value from the same experiment. The 15% cutoff was selected based on prior validation studies demonstrating robust discrimination between neutralizing and non-neutralizing samples (6). For each plate, samples from two healthy adult blood donors with no detectable levels of neutralizing auto-Abs against IFN-α2 and IFN-ω at both 10 ng/ml and 100 pg/ml were also collected to serve as negative controls. A sample from a patient with an adverse reaction to live attenuated yellow fever vaccine known to have broad neutralization status against all IFN-I conditions tested was used as a positive control.

Samples were tested for neutralization against IFN-α2 at 10 ng/ml (high), 1 ng/ml (intermediate), and 100 pg/ml (low) and against IFN-ω at 10 ng/ml (high), 1 ng/ml (intermediate), and 200 pg/ml (low). If neutralization was detected (normalized RLA [%] <15%) against at least one of these conditions, the twin was categorized as an IFN-I auto-Ab–positive twin, and this terminology and definition are used throughout the article.

Researchers conducting the IFN-I auto-Abs measurements were blinded to sample identity, zygosity, and co-twin identity. Since the study aimed to assess genetic contributions of neutralizing IFN-I auto-Abs, samples from each twin pair were separated and randomly allocated across different assay plates and experimental days to minimize potential technical batch and plate effects. This setup reduced the possibility that twin pair similarity could be due to shared experimental conditions rather than underlying biological similarity.

### Statistical analysis

#### Comparison of prevalence of IFN-I auto-Abs

Prevalences were calculated with 95% confidence intervals based on the Clopper–Pearson exact binomial method, and differences in prevalence between the Danish twin cohort and French general population–based cohort (6) were evaluated using Fisher’s exact test.

#### Concordance rates

Proband-wise concordance rate was calculated as 2C/(2C + D) among intact twin pairs, where C denotes the number of concordant twin pairs and D the number of discordant pairs (45). This measure estimates the probability that a co-twin of an affected individual is also affected. Exact 95% CIs for concordance rates were calculated using the Clopper–Pearson method. To assess whether the observed number of concordant twin pairs exceeded that expected given the low individual prevalence, the expected number of concordant pairs was calculated assuming within-pair independence. Under this assumption, the expected number was estimated as the number of twin pairs multiplied by the square of the observed individual prevalence. The probability of observing at least one concordant pair was calculated using a Poisson model.

#### Mortality analyses

Mortality analyses were first restricted to IFN-I auto-Ab–positive twins from discordant twin pairs, their IFN-I auto-Ab–negative co-twins, and matched controls selected among other intact twin pairs. Controls were matched 1:4 to IFN-I auto-Ab–positive twins and IFN-I auto-Ab–negative co-twins by cohort, sex, and birth year and were required to be alive at the time of blood sampling. A 1:4 matching ratio was chosen to improve statistical precision, with only modest gains in precision expected from additional matched controls. Follow-up started at the age of blood sampling for IFN-I auto-Ab–positive twins and negative co-twins. For matched controls, follow-up was assigned to start at the corresponding age at which their matched IFN-I auto-Ab–positive twin or IFN-I auto-Ab–negative co-twin had their blood sample taken and continued until death or end of follow-up.

Descriptive survival measures, including median survival differences within discordant twin pairs and maximum observed survival times since blood sampling, were calculated.

All-cause mortality was analyzed using Cox proportional hazards regression with age as the underlying time scale and stratification by matched sets (46). Analyses compared (1) IFN-I auto-Ab–positive twins with their matched controls, (2) IFN-negative co-twins with their matched controls, and (3) IFN-I auto-Ab–positive twins with their negative co-twins. Unadjusted and adjusted models were fitted. Adjusted models included BMI, smoking (ever vs. never), alcohol consumption (ever vs. never), and education, selected a priori as indicators of general health and socioeconomic status potentially associated with mortality, where model convergence allowed. Any deviations from the full adjustment set are indicated in the results tables. Education was classified according to the International Standard Classification of Education (ISCED 2011) and included as a binary variable (higher education yes/no). Higher education comprised ISCED levels 5–7 (short-cycle tertiary education, bachelor’s, or master’s level), while lower education comprised levels 1–4 (primary to upper secondary education). No adjustment for multiple comparisons was applied, as the mortality analyses addressed related research questions and were interpreted based on the magnitude and consistency of the effect estimates rather than statistical significance alone.

In an additional analysis, including twins from both intact and broken pairs, mortality was analyzed using Cox proportional hazards regression with age as the underlying time scale and delayed entry at the age of blood sampling. Cluster-robust standard errors were applied to account for the dependence within intact twin pairs. The adjustment set was the same as in the primary models, with the addition of sex and cohort.

Additionally, HRs for male versus female mortality were estimated to provide context for the magnitude of the IFN-related HRs. These sex-specific HRs were estimated separately (1) in the combined sample of matched controls and (2) in the full sample of intact twin pairs, using Cox regression with delayed entry at the age of blood sampling in IFN-I auto-Ab–positive twins and –negative co-twins, with adjustment as in the primary analyses and additionally for cohort.

#### Mortality during the COVID-19 pandemic

As secondary analyses, mortality during the COVID-19 pandemic period (February 1, 2020 to November 1, 2022) was estimated among twins alive at the start of the COVID-19 period with follow-up until death or end of the COVID-19 period, whichever came first. Mortality proportions during the COVID-19 period were calculated by IFN-I auto-Ab status. Cox proportional hazards regression was used with age as the underlying time scale and delayed entry at age on February 1, 2020. Analyses included twins from intact and broken pairs, with cluster-robust standard errors to account for within-pair dependence. Unadjusted and adjusted models were fitted. The adjusted model only included sex, as the other covariates used in the primary analyses were measured at blood sampling and therefore may have changed in the meantime.

Proportional hazards assumptions were assessed for all Cox models using Schoenfeld residuals. All analyses were conducted using Stata 19.5.

### Online supplemental material

Table S1 shows prevalence of individual twins with IFN-I–neutralizing auto-Abs per IFN-I subtype and concentration tested. Table S2 shows characteristics of twins from intact and broken pairs.

## Ethics statement

Written informed consent was obtained from all participants. Collection and use of biological material, and survey and registry information were approved by the Regional Committees on Health Research Ethics for Southern Denmark, and the study was registered at the Research and Innovation Organization at the University of Southern Denmark (registration number 12.067), which approves all scientific projects for the University of Southern Denmark according to the General Data Protection Regulation.

## Supplementary Material

Table S1 shows prevalence of individual twins with IFN-I–neutralizing auto-Abs per IFN-I subtype and concentration tested.

Table S2 shows characteristics of twins from intact and broken pairs.

## Data Availability

The data are available from the corresponding author upon reasonable request according to national and international General Data Protection Regulation rules and following individual data transfer agreement and material transfer agreement rules with relevant investigators.
