# Supplementary material for: Autoantibodies neutralizing type I interferons in older twin pairs: Low concordance
Source: J Hum Immun. 2026 Oct 9;2(6):e20260142. doi: 10.70962/jhi.20260142 (PMC13651747; doi:10.70962/jhi.20260142)
Supplement: Supplementary file 1 [file jhi_20260142_tables1.docx]

**Table S1.** Prevalence of individual twins with IFN-I neutralizing auto-Abs per IFN-I subtype and -concentration tested

| **Cohort** | **IFN-α2**  **10 ng/mL** | **IFN-α2**  **1 ng/mL** | **IFN-α2**  **100 pg/mL** | **IFN-ω**  **10 ng/mL** | **IFN-ω**  **1 ng/mL** | **IFN-ω**  **200 pg/mL** | **Any IFN-I** |
| --- | --- | --- | --- | --- | --- | --- | --- |
| **Twins from Intact Twin Pairs**  ***n/N***  **(%)** | 9/2636*  (0.34) | 14/2636*  (0.53) | 20/2636  (0.76) | 15/2636*  (0.57) | 22/2636#  (0.84) | 31/2636*  (1.18) | 37/2636  (1.40) |
| **Twins from Broken Twin Pairs**  ***n/N***  **(%)** | 4/634  (0.63) | 4/634  (0.63) | 7/634  (1.10) | 3/634  (0.47) | 5/634  (0.79) | 8/634  (1.26) | 13/634  (2.05) |
| **Twins with 10-year follow up**  ***n/N***  **(%)** | 1/113  (0.88) | 1/113  (0.88) | 2/113  (1.77) | 0/113  (0) | 1/113  (0.88) | 1/113  (0.88) | 3/113  (2.65) |

Samples excluded from 1(*) or 2(#) patients due to inefficient transfection efficacy in HEK293T cell luciferase assay

Abbreviations used: IFN-I, type I interferons; n/N, number positive/number tested
