# Supplementary material for: Autoantibodies neutralizing type I interferons in older twin pairs: Low concordance
Source: J Hum Immun. 2026 Oct 9;2(6):e20260142. doi: 10.70962/jhi.20260142 (PMC13651747; doi:10.70962/jhi.20260142)
Supplement: Supplementary file 2 [file jhi_20260142_tables2.docx]

**Table S2**. Characteristics of twins from intact and broken pairs.

|  | **Twins from intact pairs** | **Twins from broken pairs** | **Total** |
| --- | --- | --- | --- |
| **N** | 2,636 | 634 | 3,270 |
| **Females, n (%)** | 1,415 (54%) | 304 (48%) | 1,719 (53%) |
| **Zygosity** |  |  |  |
| **Monozygotic, n (%)** | 766 (29%) | 93 (15%) | 859 |
| **Same sex dizygotic, n (%)** | 1,096 (42%) | 270 (43%) | 1,366 |
| **Opposite sex dizygotic, n (%)** | 774 (29%) | 251 (40%) | 1,025 |
| **Unknown zygosity, n (%)** | 0 | 20 (3%) | 20 |
| **Age in years at blood sample, median (range)** | 71.4 (65.0-94.3) | 72.0 (65.0-95.7) | 71.5 (65.0-95.7) |
| **Total observed time, years** | 31,613 | 7,244 | 38,857 |
| **Median observed time (range)** | 13.8 (0.0-27.4) | 13.6 (0.2-22.9) | 13.8 (0.0-27.4) |
| **Deaths, n (%)** | 1,305 (50%) | 311 (49%) | 1,616 (49%) |
| **BMI, mean (sd)** | 26.3 (4.2) (29 missing) | 27.1 (4.4) (10 missing) | 26.5 (4.3) (39 missing) |
| **Education (higher education), n (%)** | 649 (25%) (211 missing) | 185 (29%) (42 missing) | 834 (26%) (253 missing) |
| **Alcohol consumption (drinker), n (%)** | 2,418 (92%) (27 missing) | 591 (93%) (2 missing) | 3,009 (92%) (29 missing) |
| **Smoking (smoker), n (%)** | 1,588 (60%) (34 missing) | 424 (67%) (5 missing) | 2,012 (62%) (39 missing) |
